# Coronary Calcium Scoring Using True and Virtual Non-Contrast Reconstructions on Photon-Counting CT with Differing Slice Increment: Impact on Calcium Severity Classifications

**DOI:** 10.3390/jcm14092875

**Published:** 2025-04-22

**Authors:** Marco Kaldas, Jonathan Weber, Roosha Parikh, Karli Pipitone, Karen Chau, Doosup Shin, Rick Volleberg, Ziad Ali, Omar K. Khalique

**Affiliations:** 1 St. Francis Hospital & Heart Center, Roslyn, NY 11576, USA; mkaldas@phsi.us (M.K.); jonathan.weber@chsli.org (J.W.); roosha.parikh@chsli.org (R.P.); karli.pipitone@chsli.org (K.P.); doosup.shin@chsli.org (D.S.); rick.volleberg@chsli.org (R.V.); ziad.ali@dcvi.org (Z.A.); 2 Cardiology Department, Michigan State University, East Lansing, MI 48824, USA,; 3 College of Osteopathic Medicine, New York Institute of Technology, Old Westbury, NY 11545, USA; karen.chau@hmhn.org; 4 Department of Pediatrics, Jersey Shore University Medical Center, Neptune, NJ 07753, USA

**Keywords:** coronary calcium scoring, photon-counting CT, reconstruction techniques, cardiovascular risk assessment, calcium severity classifications, imaging methodologies

## Abstract

**Background:** Cardiovascular risk assessment relies heavily on coronary calcium scoring. With an emphasis on varying slice increments, this study investigates the effectiveness of true and virtual non-contrast reconstructions on photon-counting CT. Reconstruction methods’ effects on calcium severity classifications are critical to the improvement in imaging techniques. **Methods:** This study comprised 77 participants (mean age: 63 ± 10 years, 43% female), of whom 0 had a coronary artery calcium score (CACS) of zero. In contrast to true non-contrast (TNC) 3 × 3 mm, the reconstructions included TNC 3 × 1.5 mm, virtual non-contrast (VNC) 3 × 3 mm, and VNC 3 × 1.5 mm. Agatston units served as the basis for classifications into standard clinical diagnostic categories. **Results:** High concordance between acquisition types was revealed by interclass correlation values (0.97–0.99). Comparing TNC 3 × 1.5 mm reconstructions to their VNC counterparts, misclassifications were less common (Cohen Kappa = 0.94). (K = 0.83–0.85). Significant differences in the average calcium scores and rates of misclassification highlighted the impact of reconstruction methods on precise evaluations. **Conclusions:** VNC methods demonstrated high agreement; however, with a small rate of misclassifications as compared to the gold standard method. VNC CACS may help optimize workflows but may need differing cutoffs as compared to traditional methods.

## 1. Introduction

Cardiovascular illnesses are still the primary cause of morbidity and death worldwide. Improving our knowledge and treatment of these problems requires ongoing improvements to diagnostic techniques [1]. In the field of cardiovascular diagnostics, computed tomography (CT) imaging has become a vital tool. One particularly useful method for determining the risk of coronary artery disease (CAD) is coronary artery calcium scoring (CACS) [2]. Arthur Agatson described the method of CACS in 1990 [3]. Thereafter, a robust body of literature has grown demonstrating the powerful ability of CACS to predict cardiovascular events and guide therapies [4]. To help medical professionals with risk assessment and the development of customized preventative measures, CACS is essential for detecting and measuring calcified plaques inside coronary arteries [5].

The accuracy and reproducibility of these evaluations are heavily influenced by the selected CT reconstruction methods. There have been variations in reconstruction methods for CACS. While the Agatston method was based on non-overlapping 3 mm slices (3 × 3 mm), one of the most prevalent vendors performs autoscoring using 3 mm slices with a 1.5 mm overlap (3 × 1.5 mm). Variation in slice increment is a crucial component deserving further examination [6].

Photon-counting CT (PCD-CT), a new technology promising improved spectral sensitivity, decreased imaging noise, and superior spatial resolution. CACS is frequently performed in the context of coronary CTA. Non-contrast CT used for CACS better demonstrates calcification, particularly small foci, which may be masked by iodinated contrast. With PCD-CT, it is possible to remove iodine from the CTA image and produce a virtual non-contrast (VNC) image. This allows extraction of both contrast-enhanced and non-contrast information from the same images [7].

The dynamic relationship between technology and clinical practice warrants shifts in diagnostic paradigms based on evidence from new technologies. The aims of this study were to evaluate the agreement between different slice intervals in true non-contrast (TNC) and virtual non-contrast studies and to assess the diagnostic accuracy of CACS categorization compared to the TNC 3 × 3 mm standard. We hypothesized that due to methodological differences, there would be misclassification between categories.

## 2. Materials and Methods

### 2.1. Study Design

The Standards for Reporting Diagnostic Accuracy (STARD 2015) guidelines were followed for this analysis [8]. TNC refers to non-enhanced CT, while VNC refers to the reconstructed CT subtracting iodine attenuation from post-contrast data. TNC 3 × 3 mm reconstructions, which were evaluated as a gold standard, were compared to TNC 3 × 1.5 mm, VNC 3 × 3 mm, and VNC 3 × 1.5 mm reconstructions.

### 2.2. Study Participants

For this study, 254 patients who underwent gated coronary CTA on the Siemens Naeotom Alpha PCD-CT scanner between 18 January 2023 and 30 March 2023 at a single tertiary care center were retrospectively screened. Patients with incomplete sequence acquisitions or patients with a CACS of 0 were excluded. In total, 77 patients were included in the final analysis. This study was approved by the institutional review board with a waiver of informed consent.

### 2.3. Coronary CTA Acquisition, Reconstruction, and Measurements

CACS scan and coronary CTA were acquired on the Siemens Naeotom Alpha PCD-CT scanner. CACS was acquired using 120 kvp tube voltage and flash mode and reconstructed using QR36 kernel with a quantum reconstruction setting of 2.3 mm thick slices were reconstructed using both 3 mm and 1.5 mm slice increments. Heart rate control was instituted for coronary CTA using beta-blockers and/or ivabradine for a goal heart rate of <60 bpm. Coronary CTA was acquired using 140 kvp tube voltage, sequential mode, and reconstructed using QR36 kernel with a quantum reconstruction setting of 2 and mono KEV reconstruction of 70. Virtual non-contrast images were reconstructed at 3 mm slices, using both 3 mm and 1.5 mm slice increments.

The CACS score was measured by a trained CT analyst (MK) using 3mensio software version 9.3, using the CACS module. The analyst was trained on 10–20 test sets, and results were reviewed with a level 3 certified cardiovascular CT expert (OK). This process was repeated until agreement reached 100% prior to analyzing the main study cohort.

### 2.4. Sampling Technique

During the study period, subjects who satisfied the inclusion criteria were recruited using consecutive sampling. By using this method, a representative sample of the intended group seeking coronary calcium scoring by photon-counting CT imaging was guaranteed.

Using the prescribed reconstructions (TNC 3 × 3 mm, TNC 3 × 1.5 mm, VNC 3 × 3 mm, VNC 3 × 3 mm), CACS was measured. Two research fellows trained by a level 3 cardiologist CT reader (OK), who were blind to the reconstruction methods, assessed the acquired pictures. Based on Agatston Units, the following scale was used to classify the severity of calcium: 1–99 (mildly increased risk), 100–299 (moderately increased risk), and ≥300 (moderate to severely increased risk) [9,10].

### 2.5. Statistical Analysis

The TNC 3 × 3 mm method was used as a reference standard to which the other methods were compared using standard guidelines for agreement [11]. Repeat-measures ANOVA was used to test overall calcium score differences. Overall inter-rater reliability was evaluated using Shrout-Fleiss reliability. The pairwise agreement between various reconstruction approaches compared to the TNC 3 × 3 mm method was evaluated using the Interclass (concordance) Correlation Coefficients (ICC). A correction factor was estimated using ordinary least squares linear regression models predicting TNC 3 × 3 mm CACS from the alternative reconstruction methods. Categorical agreement was also evaluated when subjects were divided into calcium score groups of 1–99, 100–300, and 300+. The pairwise agreement between reconstruction methods was measured using Cohen’s κ. Finally, sensitivity and specificity for correctly categorizing calcium score were evaluated for each pair of compared acquisitions. All analyses were performed in SAS version 9.4 (Cary, NC, USA). *p*-values <0.05 were considered statistically significant.

## 3. Results

Among the 77 subjects included, the mean age was 63 ± 10 (range: 38–82), and 41% were female. The mean BMI was 29.8 ± 6.5, and among the subjects, 60% had hypertension, 13% had diabetes 53% with hyperlipidemia, 13% with a family history of early coronary artery disease, and 78% presented with chest pain prior to their CTA. The majority of studies (84%) were of good or excellent quality. The mean ± SD (range) of calcium score using TNC 3 × 3, TNC 3 × 1.5, VNC 3 × 3, and VNC 3 × 1.5 mm reconstructions, respectively, were 300 ± 399 (11–2756), 299 ± 392 (16–2681), 279 ± 362 (3–2208), and 276 ± 359 (2–2194), respectively (repeat-measures ANOVA *p* < 0.001).

**Figure 1:**
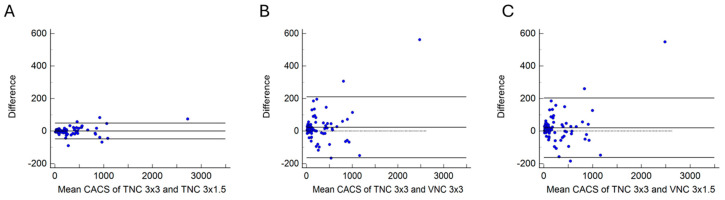
(**A**) Bland-Altman figure for TNC 3 × 3 vs. TNC 3 × 1.5. (**B**) Bland-Altman figure for TNC 3 × 3 vs. VNC 3 × 3. (**C**) Bland-Altman figure for TNC 3 × 3 vs. VNC 3 × 1.5.

The overall agreement among all measurements was near perfect (ICC = 0.98 using Shrout-Fleiss reliability). The pairwise concordance comparing each method to the TNC 3 × 3 mm standard was also near-perfect, with the TNC 3 × 1.5 mm reconstruction method performing slightly better than the two other methods (Table 1 and Figure 1A–C).

**Figure 2:**
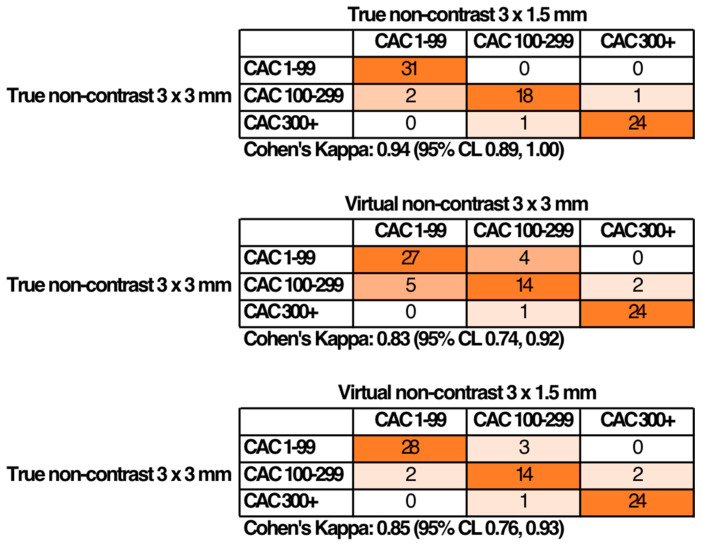
Coronary calcium scoring categories in different reconstruction methods.

Upon dividing subjects into calcium score categories, agreement between categories was excellent (Figure 2). Upon comparing TNC 3 × 1.5 mm reconstructions to the gold standard TNC 3 × 3 mm, the results showed that for CACS 1–99, there were 31 instances of substantial agreement (Cohen’s κ = 0.94), two instances of slight disagreement (for CACS 100–299), and perfect agreement (for CACS 300+) (Figure 2). The comparison of TNC 3 × 3 mm and VNC 3 × 3 mm reconstructions revealed 27 instances of agreement for CACS 1–99, five instances of disagreement for CACS 100–299, and perfect agreement for CACS 300+ (Cohen’s κ = 0.83) (Figure 2). Similarly, when TNC 3 × 3 mm was compared to VNC 3 × 1.5 mm, there were 28 instances of agreement for CACS 1–99, two instances of disagreement for CACS 100–299, and perfect agreement for CACS 300+ (Cohen’s κ = 0.85).

Linear regression demonstrated a small correction factor that could be utilized to estimate the TNC 3 × 3 mm calcium score (Table 2). Parameter estimates (β_reconstruction_) were slightly higher for VNC reconstructions, whereas every AU increase in calcium score was associated with a 1.015 AU, 1.082 AU, and 1.075 AU increase in predicted TNC 3 × 3 mm score for TNC 3 × 1.5 mm, VNC 3 × 3 mm, and VNC 3 × 1.5 mm reconstructions, respectively. The direction of the associations and Bland-Altman plots (Figure 1) demonstrate slight overall underestimation of TNC 3 × 3 mm reconstruction-based CACS by the alternative methods.

Among the reconstruction modalities, TNC 3 × 1.5 mm overall had the best sensitivity and specificity as compared to TNC 3 × 3 mm (Table 3). Additionally, the reconstruction methods overall more successfully identified/ruled out calcium when the score was higher (300+) compared to lower calcium categories (0–99 or 100–299).

## 4. Discussion

The current analysis is the largest PCD-CT study to date comparing CACS using TNC and VNC techniques.

The main findings of this study are as follows:

(1) There was excellent overall concordance between calcium scores using the 4 reconstruction methods studied.
(2) Calcium scores from VNC were lower than those from TNC.
(3) Correct classification of calcium risk category for true non-contrast 3 × 1.5 mm was very high, and for virtual non-contrast cases was moderate to high.
(4) Slice interval (overlap) did not appear to play a significant role in misclassification.

### 4.1. Formatting of Mathematical Components

Agatston calcium scoring has been the gold standard for CT-based patient screening risk assessment for decades. While there have been limited medical therapies available for plaque stabilization and treatment in the past (mainly aspirin and statins), the number of therapies has increased over time, and newer trials and registries such as the DECODE study [12], DECIDE registry (unpublished), and TRANSFORM trial [13] will begin to explore differential medical therapy recommendations based on quantitative plaque burden. While PCD-CT technology has only come into commercial usage in the past few years, the number of scanners being used is exponentially increasing, and it appears to be the future gold standard in cardiac CT. Thus, it is critical for early adopters to study techniques and investigate efficiencies that will be needed for the future.

### 4.2. Prior Studies on CAC Scoring Using Spectral CT and PCD-CT

Several prior studies have evaluated CAC scoring using VNC images on either spectral CT or PCD-CT and have been very consistent in showing that CACS on VNC images underestimated that of TNC.

Langenbach et al. demonstrated that TNC images at 2.5 mm slice thickness most closely agreed with VNC images at 2.5 mm slice thickness without significant difference in CACS but were different from VNC images based on 0.9 mm slice thickness using dual-layer spectral CT [1]. Yang et al., Nadjiri et al., and Gassert et al. demonstrated an underestimation of TNC CACS by VNC using dual-layer spectral CT [2,14,15]. Song et al. demonstrated similar findings to Yang, using dual-energy spectral CT [16].

Symons et al. demonstrated a higher calcium-to-soft tissue signal ratio using phantoms and a better agreement with standard versus low radiation in vivo scans with protype PCD-CT as compared to Energy-Integrating Detector (EID)-CT, which suggests more consistent image quality across tube output for PCD-CT [17]. Mergen et al. demonstrated an underestimation of TNC CACS by VNC CACS at various iterative reconstruction and virtual monoKEV levels using dual-source PCD-CT [18]. Sharma, Emrich, and Braun et al. had similar findings to Mergen et al., also using dual-source PCD-CT [19,20,21]. All 4 of these investigator groups used 3.0 × 1.5 mm slice reconstructions for CACS.

### 4.3. Current Study

The current analysis confirms that VNC reconstructions systematically underestimate the CACS as compared to TNC. Whereas VNC mean CACS were lower than TNC, correlation was excellent. Thus, a correction factor is needed when using VNC to align with currently accepted CACS classifications (Table 2). One factor that is underrepresented in the current literature and guidelines is the variation in slice increment. An additive finding of our study to the current literature was that slice increment did not play a role in variation of CACS, as evidenced by the lack of difference in CACS within the TNC and VNC categories, respectively. Thus, it is reassuring that using a manufacturer-recommended 3 × 1.5 mm increment or the more traditional 3 × 3 mm settings produces the same CACS. Whereas there are various other parameters that can be modified, our goal was to study TNC vs. VNC and slice increment parameters while keeping other parameters, such as KEV, kernel, and iterative reconstruction level, consistent so as to not introduce other variabilities into the analysis.

Leading up to the 2021 ACC/AHA chest pain guidelines [22], there has been a significant year-over-year growth in the number of coronary CTAs in the United States, and the growth has been accelerating since a class 1 indication was reported in the guidelines. Recent analyses of Medicare data demonstrated an 84% increase in utilization of cardiac CTA from 2010 to 2019 [23], with a 77% increase in the number of available physicians who provide CT services [24] and a further 60% increase in utilization from 2019 to 2023 [25]. A non-contrast calcium score typically precedes the coronary CTA but adds slightly more time due to the need for 2 separate scans. With increased need for efficiency, VNC reconstruction is a possible novel use of PCD-CT as a more efficient method to provide Agatston calcium scoring and angiographic information from the same dataset. In addition, calcium scoring is being used in the new field of CT-guided PCI as one aspect of PCI planning to determine the need for calcium modification strategies [26].

An implication of our analysis in the setting of these large imaging volume increases is the ability to streamline workflows to potentially avoid the need for a separate CACS score prior to coronary CTA. Our group has previously demonstrated the ability of PCD-CT to more accurately identify coronary stenosis needing intervention regardless of calcium score [27]. This may obviate the necessity to perform separate CACS from a coronary CTA triaging standpoint. Thus, in a busy practice performing 20 coronary CTAs in a day, even a few minutes per case saved performing and assessing the TNC CACS can lead to significant increases in scanning efficiency and the ability to perform extra scans. Although not specifically studied here in a comparison cohort, an additional theoretical benefit to the patient could be the elimination of the small amount of radiation associated with the separate TNC CACS exam. The VNC CACS reconstruction does not require additional radiation to the standard PCD-CT coronary CTA.

### 4.4. Limitations

The current study was a single-center, retrospective study with a relatively small sample size, limiting our ability to generalize our results to different populations. Nevertheless, it is the largest such study to date using PCD-CT, and our sampling method adequately ascertained a cross-section of subjects typically referred from our catchment area. As this analysis was based on calcium scoring, which is only one aspect of plaque morphology, there was no assessment of non-calcified plaque. This study was performed on a single PCD-CT scanner; further generalizations to different scanners and protocols are limited, and confirmations may be beneficial. Finally, our linear model-based correction factors require additional external validation before they can be implemented in clinical workflows. Additional study is required to highlight how motion artifacts and changes in calcium density or plaque morphology may alter the concordance between reconstruction methods.

## 5. Conclusions

VNC calcium scoring with a correction factor may be used in place of TNC methods, leading to possibilities of more efficient scanning and workflows.

## Figures and Tables

**Table 1:** Mean difference and inter-class correlation between TNC 3 × 3 mm reconstruction versus other reconstructions.

Reconstruction Comparison	Mean 1	Mean 2	Mean Diff	Inter-Class Correlation (95% Confidence Interval)
TNC 3 × 3 vs. TNC 3 × 1.5 mm	300 ± 399	299 ± 392	0.8 ± 25	0.998 (0.997 to 0.999)
TNC 3 × 3 vs. VNC 3 × 3 mm	300 ± 399	276 ± 359	23.4 ± 95	0.967 (0.95 to 0.978)
TNC 3 × 3 vs. VNC 3 × 1.5 mm	300 ± 399	279 ± 362	20.3 ± 93	0.969 (0.953 to 0.979)

**Table 2:** Slope parameters to predict gold-standard TNC 3 × 3 mm from other reconstruction methods.

Reconstruction Method	β_0_ *	SE of β_0_	β_reconstruction_	SE of β_reconstruction_	β_reconstruction_ *p*-Value	R^2^
TNC 3 × 1.5 mm	−3.79369	3.54552	1.01497	0.00718	<0.0001	0.996
VNC 3 × 3 mm	0.08412	13.37829	1.08184	0.02945	<0.0001	0.948
VNC 3 × 1.5 mm	−1.58745	13.15751	1.07538	0.02871	<0.0001	0.950

Abbreviations: TNC = true non-contrast; VNC = virtual non-contrast; β_0_ = y-intercept; SE = standard error; β_reconstruction_ = slope of the reconstruction parameter of interest. Info: The model takes the form of Y_TNC 3 × 3_ = β_0_ + β_reconstruction_ * X_reconstruction_, where X_reconstruction_ is the value of the CAC using the reconstruction method of interest.

**Table 3:** Accuracy of modified TNC and VNC reconstructions to correctly predict strata of coronary artery calcium score compared to the TNC 3 × 3 mm standard.

	Specificity	Sensitivity
TNC 3 × 1.5		
1 to 99	0.95	1
100 to 299	0.98	0.86
300+	0.98	0.96
VNC 3 × 3		
1 to 99	0.88	0.87
100 to 299	0.91	0.67
300+	0.95	0.96
VNC 3 × 1.5		
1 to 99	0.88	0.9
100 to 299	0.93	0.67
300+	0.95	0.96

## Data Availability

The data presented in this study are available on reasonable request from the corresponding author.

## Abbreviations

CACS	coronary artery calcium score
CAD	coronary artery disease
CT	computed tomography
TNC	true non-contrast
VNC	virtual non-contrast
CTA	CT angiography
